# Limited Exchange Transfusion Can Be Very Beneficial in Sickle Cell Anemia with Acute Chest Syndrome: A Case Report from Tanzania

**DOI:** 10.1155/2018/5253625

**Published:** 2018-06-21

**Authors:** Clara Chamba, Hamisa Iddy, Erius Tebuka, Furahini Tluway, Elisha Osati, Neema Budodi, Collins Meda, Mbonea Yonazi, Anna Schuh, Lucio Luzzatto, Julie Makani

**Affiliations:** ^1^Sickle Cell Programme, Department of Hematology and Blood Transfusion, Muhimbili University of Health and Allied Sciences, Dar es Salaam, Tanzania; ^2^Department of Pathology, Catholic University of Health and Allied Sciences, Mwanza, Tanzania; ^3^Haematology Unit, Department of Internal Medicine, Muhimbili National Hospital, Dar es Salaam, Tanzania; ^4^Department of Oncology, University of Oxford, Oxford, UK

## Abstract

Acute chest syndrome (ACS) is a life-threatening complication of sickle cell disease (SCD) with blood transfusion an integral part in its management. Red cell exchange (RCE) transfusion is usually regarded as preferable to top-up transfusion, because it reduces the proportion of Hemoglobin (Hb) S while at the same time avoiding circulatory overload. Despite its obvious benefits, RCE is underutilized, particularly in low-resource settings which may be due to scarcity of blood products and of expertise in carrying out exchange transfusion. We report on a young woman with SCD with severe ACS who responded promptly and dramatically to a RCE of only 0.95 L (instead of the recommended 1.4 L) and had in the end an HbS level of 48% (instead of the recommended level below 30%). Limited RCE resulted in significant clinical improvement. We suggest that limited RCE may be of benefit than no RCE in SCD patients with ACS, particularly in settings where RCE is not available.

## 1. Introduction

Sickle cell disease (SCD) is a genetic disorder affecting red blood cells with a high prevalence in Africa and a high morbidity and mortality [1]. Among complications of SCD, the acute chest syndrome (ACS) is a potentially life-threatening one; it presents with chest pain, fever, cough, dyspnoea; tachypnoea, decreased peripheral oxygen saturation, and pulmonary infiltrate(s) on chest X-ray. ACS is a frequent cause of hospitalization in SCD patients [2], and it may account for up to 25% of SCD-related deaths. The mortality risk due to ACS has been estimated to be four times higher in adults compared to children [3, 4].

The management of ACS aims to support the patient through supplemental oxygen therapy, appropriate analgesics, hydration, and antibiotics [5]. Blood transfusion is indicated in order to increase the oxygen carrying capacity of the blood and reduce the proportion of red cells susceptible to sickling [6, 7]. However, a simple (so-called “top-up”) transfusion may also immediately cause increased blood viscosity and an increased circulating blood volume, which may increase the load on the heart. Furthermore, recurrent blood transfusions contribute to iron overload [8]. For these reasons, exchange blood transfusion or red cell exchange (RCE) has been introduced and is recommended for the management of ACS [3, 5].

RCE is nowadays frequently carried out by the use of automated equipment; however, it can be also carried out manually with minimal equipment [9]. We report on a critically ill young woman with SCD and ACS who was successfully managed by manual partial exchange transfusion in our hospital.

## 2. Case Presentation

A 17-year-old female student with known SCD (HbSS), on folic acid (FA) since early childhood and on hydroxyurea (HU) 500 mg/day for the past 5 years, was admitted to the medical ward with difficulty in breathing, persistent fever, chest pain, dry cough, and general body malaise for four days prior to admission. Her previous history included a stroke 5 years ago (from which she had made a good recovery) and avascular necrosis of the right hip joint. Prior to this admission, her last transfusion had been 5 years ago.

On physical examination, the patient was febrile (38°C), pale, mildly jaundiced, tachypnoeic, and dyspnoeic with no lower limb edema. Her respiratory rate was 50 breaths/min, pulse rate was 124 bpm, and blood pressure was 124/64 mmHg. Her oxygen saturation (SPO_2_) was 65% on room air and 100% on oxygen. On chest examination, there were reduced breath sounds in the right base. She had no palpable enlargement of the liver or spleen.

Hemoglobin was 84 g/L, leukocytosis of 19.8 × 10^9^/L, with a predominance of neutrophils (12.4 × 10^9^/L), and platelets of 535 × 10^9^/L (Table 1). From previous records, her steady-state hemoglobin was about 70 g/L. Urine culture revealed no bacterial growth after 24 hours; renal function tests and serum electrolytes were within the normal range (Table 2). A chest X-ray showed right-sided consolidation, bilateral mid-zone changes, and cardiomegaly. A diagnosis of ACS was made, but pneumonia and septicemia could not be excluded. The patient was placed on oxygen supplementation and was started on IV cefoperazone/sulbactam 2 g·bd, IV normal saline (NS) 3 L/24 hours, and oral ibuprofen; hydroxyurea and folic acid were continued. Pain was managed by syrup morphine 5 mg 4 hourly. Two units of packed red cells were cross-matched and transfused.

After 7 days, the difficulty in breathing worsened, and the patient was transferred to a High Dependency Unit (HDU). Pleurocentesis was performed, and a yellowish frothy fluid was aspirated and taken for culture, biochemistry, and cytology. Culture revealed no bacterial growth after 72 hours, and the biochemistry results were normal (glucose 0.5 mol/l and protein 35 g/L). Cytology showed presence of neutrophils, histiocytes, and lymphocytes. The results of these tests as well as serum adenosine deaminase (22.7 IU/L) and ESR (10 mm/hr) were regarded as not compatible with pulmonary tuberculosis. Viral serology for hepatitis B surface antigen, hepatitis C antibody, and ELISA for HIV I and II were all negative. Oral co-trimoxazole was added to her therapy as a cover for possible atypical pneumonia.

**Figure 1:**
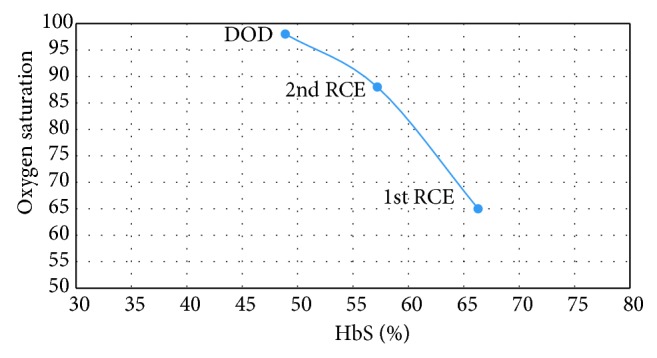
Oxygen saturation (%) versus HbS (%). DOD = day of discharge.

Despite improvement in the right basal consolidation on the repeat chest X-ray, the bilateral mid-zone changes were still present and there was no improvement in her clinical condition. On day 3 in the HDU, the patient's condition deteriorated: her RR was 72/min with PR of 124 b/min and SPO_2_ ranging between 60 and 66% on room air. The patient was prepared for manual RCE. Prior to exchange transfusion, the hemoglobin was 95 g/L (Table 1) and HbS quantified by HPLC was 66.4% (Figure 1), as a result of the recent blood transfusion.

RCE was performed in two steps 20 hours apart. In the first step, a total of 500 ml of the patient's blood was replaced by 250 ml of normal saline (NS) and 250 ml of packed red cells. The patient developed a febrile nonhemolytic transfusion reaction which was managed by IV paracetamol 1 g. After this initial step, the Hb was 98 g/L and HbS was 57.2% (Figure 1). 12 hours after the RCE, the patient was afebrile, and the SPO_2_ was 83–88% on room air. In the second step, 450 ml of the patient's blood was exchanged with 250 ml of NS and 200 ml of packed red cells. In total, 950 ml were replaced, corresponding to an estimated 30% of the total blood volume.

Twelve hours after the second RCE, the hemoglobin was 92 g/L and HbS was 48.9%. The patient was afebrile, with oxygen saturation of 97–100% on room air (Figure 1), RR of 28–30/min and pulse rate of 90 bpm. The patient was weaned off oxygen and 48 hours after exchange, the patient was moved back to the general ward, from where she was discharged three days later. A week later, she attended the outpatient hematology clinic, where she was found to be in good condition, with stable vital signs and a close to normal blood count. In her most recent follow-up (4 months after RCE), she is doing well and has no complaints. She is currently on folic acid 5 mg daily and hydroxyurea 500 mg daily.

## 3. Discussion

This patient with SCD met the clinical and radiological criteria for the diagnosis of ACS, and on RCE, she had full clinical resolution of ACS. This result was obtained in spite of the fact that only 0.95 L of blood was exchanged, instead of the recommended amount of 1.4 L [9]. Furthermore, after RCE, her HbS level was 48% and did not reach the commonly recommended <30%.

With respect to the clinical benefit observed in this patient, we offer the following comments:

1. Although the patient had marked tachypnoea and marked desaturation, she did tolerate a 6-day wait before the exchange; a patient with a more severe ACS might not have survived that long. Therefore, we classify her ACS as moderately severe.
2. In spite of this delay, we have no doubt that RCE was crucial in resolving the patient's ACS; indeed, her respiratory rate started decreasing and her blood oxygen saturation started improving soon after the RCE was started. Both parameters were completely normal by the time RCE was over (Figure 1).
3. In spite of the eventual good outcome, RCE should have been carried out earlier, before the clinical state became life threatening.
4. Although we are not aware of any evidence basis for the currently recommended volume of RCE or target HbS percentage, we are not suggesting a change to current recommendations. However, we note that too many times RCE is not carried out due to “lack of equipment” or shortage of blood. This case illustrates that the equipment needed is minimal and that even 2 blood units together with equivalent amount of normal saline may be sufficient to produce a dramatic clinical benefit.

The result reported here is certainly not unique, as in various centers experienced pediatric cytapheresis teams have performed partial RCE with a target posttransfusion HbS of 60–70% (rather than <30%) [10]; but, we do not know how often this is practiced. In order to provide an evidence base for amended recommendations on RCE in SCD with ACS, a randomized clinical trial would be ideal, but not easy to carry out. In the meantime, our patient may be one example demonstrating that a limited RCE is better than no RCE; sometimes, it might be life-saving.

## Figures and Tables

**Table 1:** Serial full blood picture results.

Date	04/07 (ADM)	13/07 (post-BT 2 units)	17/07	18/07 post-RCE 1	21/07 post-RCE 2	04/08 CV 1	03/11 CV 2	Normal range
TWBC (×10^9^/L)	19.8	44.8	26.9	28.1	24.8	07.4	13.1	4.0–10.0
aNeut (×10^9^/L)	12.4	37.4	18.2	20.6	22.1	03.4	06.0	2.0–6.9
aLym (×10^9^/L)	04.1	04.3	07.1	04.8	01.0	03.5	05.4	0.6–3.4
RBC (×10^12^/L)	02.3	02.8	02.9	03.2	03.3	03.2	02.3	3.8–4.8
HCT (%)	23.5	27.8	29.0	29.9	29.6	29.5	22.3	36.0–46.0
Hb (g/L)	84.0	93.0	95.0	98.0	92.0	90.0	76	120–150
MCV (fl)	101.0	98.0	102.0	92.4	88.6	92.0	98.3	83.0–99.0
MCH (pg)	35.8	33.0	33.2	30.3	29.3	28.2	33.3	21.0–32.0
MCHC (g/dl)	35.5	33.7	32.6	32.8	33.1	30.6	33.9	31.5–34.5
Platelets (×10^9^/L)	535	525	656	231	729	607	436	150–410
Reticulocytes (%)	—	—	—	20	—	—	—	—

ADM = on admission; BT = blood transfusion; RCE = red cell exchange; CV = clinic visit.

**Table 2:** Clinical chemistry.

Date	13/07	27/07	Normal range
Blood urea nitrogen (mmol/l)	1.3	1.1	2.5–6.7
Creatinine (*µ*mol/l)	18.0	44.0	50.4–99.1
ALT (U/L)	11.0	—	0–55
AST (U/L)	23.0	—	5–34
Potassium (mmol/l)	3.8	7.2	3.5–5.1
Sodium (mmol/l)	137.0	136.0	136–145
Chloride (mmol/l)	110.0	—	98–107
Calcium (mmol/l)	—	2.10	2.10–2.55
Magnesium (mmol/l)	—	0.81	0.66–1.07
Phosphorus (mmol/l)	—	1.18	0.74–1.52
